# Potential Prognostic Impact of Dopamine Receptor D1 (*rs4532*) Polymorphism in Post-stroke Outcome in the Elderly

**DOI:** 10.3389/fneur.2021.675060

**Published:** 2021-06-30

**Authors:** Hae-Yeon Park, Youngkook Kim, Hyun Mi Oh, Tae-Woo Kim, Geun-Young Park, Sun Im

**Affiliations:** ^1^Department of Rehabilitation Medicine, Seoul St. Mary's Hospital, College of Medicine, The Catholic University of Korea, Seoul, South Korea; ^2^Department of Rehabilitation Medicine, Yeouido St. Mary's Hospital, College of Medicine, The Catholic University of Korea, Seoul, South Korea; ^3^Department of Rehabilitation Medicine, National Traffic Injury Rehabilitation Hospital, Yangpyeong, South Korea; ^4^Department of Rehabilitation Medicine, College of Medicine, Seoul National University, Seoul, South Korea; ^5^Department of Rehabilitation Medicine, Bucheon St. Mary's Hospital, College of Medicine, The Catholic University of Korea, Seoul, South Korea

**Keywords:** single nucleotide polymorphism, swallowing, aged, prognosis, stroke, elderly

## Abstract

**Background:** Single-nucleotide polymorphisms (SNPs) may affect post-stroke motor recovery, and some SNPs have been implicated in swallowing disturbances after stroke. Certain SNPs may also have altered influences according to different age.

**Objective:** This *post-hoc* study investigated whether SNPs have different effects on dysphagia recovery between the elderly vs. young stroke patients.

**Methods:** Analysis was conducted from a previous study including 218 stroke subjects with dysphagia. They were stratified into two groups, aged <65 and aged ≥65 years. The primary outcome was persistence of nil per mouth (NPM) at 3 months post-stroke onset. Association between outcome and nine different SNPs were investigated.

**Results:** The elderly group (50%, *n* = 103) showed poorer swallowing outcomes than the young group. The minor allele of the dopamine receptor D1 (DRD1, *rs4532*) polymorphism showed potential association (*p* = 0.022) with an increased risk of NPM at 12 weeks post-stroke in the elderly, both in the additive (OR, 2.94; 95% CI, 1.17–7.37) and dominant models (OR, 2.93; 95% CI, 1.04–8.23) but did not reach statistical significance after Bonferonni correction. Logistic regression analysis showed that in those aged ≥65 years, models including the minor allele of *rs4532* predicted the risk of the poor outcome with good accuracies even after adjustment of clinical factors, such as previous pneumonia episodes (AUROC, 0.86; 95% CI, 0.79–0.93) or the National Institutes of Health Stroke Scale (AUROC, 0.82; 95% CI, 0.67–0.92). In contrast, those aged <65 years seemed not to be affected by the presence of the *rs4532* polymorphism, and models that included intubation history (AUROC, 0.81; 95% CI, 0.73–0.90) or previous pneumonia episodes (AUROC, 0.77; 95% CI, 0.68–0.87) showed modest levels of accuracies in predicting NPM at 12 weeks poststroke.

**Conclusions:** Our study suggests a possible association between the *rs4532* and post-stroke swallowing recovery, primarily in those aged ≥65 years. Certain SNPs may lead to less favorable outcomes in the elderly. The gene–age interaction should be considered in post-stroke swallowing recovery.

**Clinical Trial Registration:** https://www.clinicaltrials.gov, Unique identifier [NCT03577444].

## Introduction

Dysphagia is common in elderly populations, accounting for about 15% of the geriatric population (1). Swallowing impairment in neurodegenerative diseases, such as Parkinson's disease, may begin in the early stage and may contribute to the increased mortality (2). Neurogenic dysphagia due to acquired brain lesions, such as infarction or hemorrhage, is also one of the leading causes of chronic disability. Dysphagia can be observed in about 40–60% of post-stroke patients (3), and about 20–30% of them might suffer from recurrent aspiration pneumonia (4); one of the leading causes of post-stroke mortality. Due to the high mortality resulting from aspiration pneumonia, there have been studies to discover predicting factors associated with dysphagia. In early-stage Parkinson's disease, excessive daytime sleepiness has been known to be related with dysphagia, and in predicting post-stroke pneumonia, stroke severity and old age are known as poor prognostic factors (5, 6).

Previous studies (7–13) have reported that genetic polymorphisms may affect the outcomes of stroke. For example, the Val66Met polymorphism in the brain-derived neurotrophic factor (BDNF) gene has been linked to poor motor recovery after stroke (7). On the other hand, the Val/Val allele in the catechol-O-methyl transferase (COMT) gene is associated with higher motor functions in post-stroke patients (8). Others have pointed that during the first-month post-stroke, the Apolipoprotein E (ApoE) ε4 polymorphism may be associated with poor recovery (12).

The effects of single nucleotide polymorphism (SNP) may manifest differently in the elderly age groups. In 2008, Lindenberger et al. (14) explained that the effects of common genetic variation on brain and behavior may be amplified with age. In younger individuals, differences in neural reserve result in small variations in cognitive performance, whereas with increased age, even a small perturbation in neural reserve may result in a large variation in cognitive performance. In other words, genetic variability may affect performance more strongly with increased age.

In line with this theory, the effect of age on stroke recovery has also been highlighted in some studies. Functional parameters such as Barthel Index (BI) and Rankin score showed a difference between those ≥55 years old and those <55 years old with ischemic stroke (15). In addition, the elderly stroke are at increased risk of dysphagia and poor swallowing recovery than the younger age groups (16, 17). Although there have been studies on SNPs' effect on post-stroke recovery (7, 8, 11, 12) and the possible gene interaction between dysphagia prevalence in the geriatric population (10), reports are still scarce whether certain SNPs may exert different influences to affect post-stroke dysphagia recovery between the elderly and the young individuals. After stroke, identifying those at increased risk of poor swallowing recovery through feasible biomarkers is important to prevent respiratory complications, and SNPs could be one of the beneficial biomarkers.

In light of recent literatures (15, 18, 19) that show SNPs may have variable influences specifically in the elderly, who are already vulnerable to swallowing disturbance and poor recovery, we hypothesized that swallowing in this age group would be more heavily influenced by genetic variations than younger individuals after stroke. Taken into consideration that neuroplasticity may exert differently in different age groups, whether these same SNPs could lead to different outcomes in the elderly after a stroke has yet to be validated.

Therefore, in this study, we performed a *post-hoc* analysis from a previous prospective trial (20) with aims to explore whether certain genes would show an increased association of poor outcome in the elderly stroke patients with swallowing disturbance. In that previous trial, we have assessed several SNPs and followed-up patients up to 3 months post-stroke. To address this issue, we reanalyzed the SNP effects between the two age groups, aged <65 (young) and ≥65 (elderly) years and assessed whether specific SNP polymorphism in the elderly could adversely affect swallowing recovery after a stroke. The primary outcome was the persistence of nil per mouth (NPM) at 3 months (12 weeks) post-stroke onset.

## Methods

### Participants

This study is a *post-hoc* analysis, and the details of participants and the rationale for the sample size are described in the previous report (20). In summary, the study involved patients with previous episode of stroke for the first time from August 2018 to July 2019 and presented dysphagia symptoms. Those with presence of other asymptomatic lesions at the time of the initial examination were not included in the study. This study was undertaken with written informed consent from all patients or legal guardians for genetic testing, and the study was approved by the institutional review board of the Catholic Medical Center (HC17TNDI0049). All methods were performed in accordance with the relevant guidelines and regulations. The primary endpoint was the persistence of NPM status with total tube feeding by 12-weeks post-stroke onset, which was defined as the poor outcome group.

### Age Stratification

In order to evaluate whether genetic polymorphisms contributed to different outcomes according to age, between the younger and elderly individuals, the cohort was stratified into two groups aged <65 (young) and ≥65 (elderly) years. This age limit was chosen based on recent reports that first, swallowing disorders, on average, affected individuals aged around 65 years old (21), and second, that age above 65 was linked to poor prognostication in post-stroke swallowing recovery (22).

### Genotyping

Around 2 cm^3^ of whole blood was obtained from all patients who consented to genotyping with TaqMan SNP Genotyping Assays. SNP genotyping assays were performed with real-time polymerase chain reaction systems. Allelic discrimination was performed. Further details of genotyping and analysis are provided in our previous study (20). SNPs were evaluated for COMT (*rs4680* and *rs165599*) (10, 23), BDNF (*rs6265*) (24), and dopamine receptors (DRD1; *rs4532*, DRD2; *rs1800497*, and DRD3; *rs6280*) (25), which were previously reported to be associated with post-stroke motor recovery. ApoE (*rs429358* and *rs7412*) (12, 26) was evaluated for its association with poor stroke recovery and dysphagia in geriatric population. Finally, interleukin 1 receptor antagonist gene (*rs4251961*) (27), which may predict stroke outcome early after stroke, was also assessed.

### Swallowing Assessment

Swallowing assessments were carried out using the Modified Barium Swallow Impairment Profile (MBSImP) (28), Mann Assessment of Swallowing Ability (MASA) (29), Functional Oral Intake Scale (FOIS) (30), and Gugging Swallowing Screen (GUSS) (31). By performing the videofluoroscopic swallowing study (VFSS), MBSImP scores and Penetration-Aspiration Scale (PAS) (32) were collected. Quality of life associated with dysphagia was measured using Eating Assessment Tool (EAT-10) questionnaires (33). The VFSS was performed at baseline and 3 months after stroke onset. All swallowing assessments were done by a certified specialist.

### Functional Assessment

Stroke severity was evaluated with the National Institutes of Health Stroke Scale (NIHSS) (34) at baseline. Patients' mobility and daily life activity were evaluated using the modified Rankin Scale (mRS) (35), Functional Ambulatory Category (FAC) (36), and Modified Barthel Index (MBI) (37). Other functional evaluations included the Mini-mental State Examination (MMSE) (38) and Berg balance scale (BBS) (39).

### Aspiration Pneumonia Incidence

During the follow-up period, any episodes of aspiration pneumonia within a 3-month period post-onset was recorded. Aspiration pneumonia was defined by the presence of respiratory symptoms with a temperature exceeding 38°C, leukocytosis, and infiltration confirmed by chest radiography warranting the use of antibiotics (40).

### Statistical Analysis

All statistical analyses were performed using SAS Version 9.4 (SAS Institute, Cary, NC, USA) and a *p*-value below 0.05 was considered statistically significant. Due to the evaluation of multiple genes, Bonferonni correction was applied in genetic evaluation and significance level for genetic testing was determined as *p* < 0.05/8 = 0.006. Sample size calculation method is described at a previous study on BDNF (20), calculated by prevalence of poor recovery set at 20%, minor allele frequency set at 30%, and relative risk of 3.138 with the required sample to be 195. Categorical variables are expressed as numbers, including percentages, while continuous variables are expressed as means ± standard deviations, or medians including interquartile ranges. A Hardy–Weinberg equilibrium using an exact test was used to assess SNP frequency. Multiple inheritance models (additive, dominant, and recessive) were used to assess the association between each SNP and risk of poor swallowing outcomes. Because of the previous study (20) that showed no increased risk with poor swallowing outcomes when all ages were considered together as one group, and in order to further assess whether SNPs would have different influences in elderly compared to young populations in the analysis, a multiple inheritance model analysis was performed with subjects separated into two age groups, over, and under 65 years of age. For each genetic polymorphism, odds ratios (ORs) and 95% confidence intervals (CIs) were evaluated by logistic regression analysis, separately in the different age groups. If certain SNPs were identified to be potentially associated with poor outcome, these SNPs were added to a final logistic regression model that also included clinical variables increasing the ORs of poor outcome identified from the univariable regression analysis. Because older age and gender are known to be poor prognosis of post-stroke dysphagia (41), age, and gender were included as fixed variables for all models.

## Results

### Patient Characteristics

A total of 206 patients had clinical data and genotype analyses available. The enrollment process of the cohort study is described in detail elsewhere (20). The elderly group (*n* = 103) showed significantly more patients with ischemic stroke, with more afflicted with aspiration pneumonia and worse baseline scores in the MMSE and BBS than the young-age group. However, no group differences in the initial stroke location or severity, as assessed by the NIHSS, were observed. Also, no baseline differences in other functional or swallowing parameters were observed. Further baseline demographics of the patients are presented in Supplementary File 1.

### Swallowing and Functional Outcomes in the Two Age Groups

The swallowing and functional outcomes between the poor vs. good outcome group (i.e., recovery from NPM status by 12 weeks) in the two age groups are shown (Table 1). Overall, the poor outcome group in both the elderly and young group showed higher NIHSS scores with worse functional and swallowing performance at baseline with increased incidence of pneumonia. Those from the young age group showed a larger number of patients with bilateral brain lesions or with intracerebral hemorrhage in the poor outcome group.

**Table 1:** Swallowing and functional outcomes of the poor vs. good outcome group in the two age groups.

	**Age** **<** **65 years (*****n*** **=** **103)**		***p*-value**	**Age** **≥** **65 years (*****n*** **=** **103)**		***p*-value**
	**Good outcome (*n* = 66)**	**Poor outcome (*n* = 37)**		**Good outcome (*n* = 60)**	**Poor outcome (*n* = 43)**	
Sex (male)	48 (72.7)	21 (56.8)	0.098	39 (65.0)	28 (65.1)	0.990
NIHSS	10.9 ± 6.8	17.5 ± 6.7	<0.0001^*^	10.9 ± 7.0	15.8 ± 6.6	0.001^*^
Stroke type			0.043^*^			0.381
Infarction	34 (51.5)	11 (29.7)		44 (73.3)	30 (69.8)	
Hemorrhage	31 (47.0)	23 (62.2)		14 (23.3)	13 (30.2)	
Both	1 (1.5)	3 (8.1)		2 (3.3)	0 (0.0)	
Lesion location			0.561			0.934
Supratentorial	49 (74.2)	24 (64.9)		45 (75.0)	31 (72.1)	
Infratentorial	14 (21.2)	10 (27.0)		14 (23.3)	11 (25.6)	
Multiple	3 (4.5)	3 (8.1)		1 (1.7)	1 (2.3)	
Lesion side			0.001^*^			0.295
Right	26 (39.4)	7 (18.9)		25 (41.7)	15 (34.9)	
Left	32 (48.5)	14 (37.8)		29 (48.3)	19 (44.2)	
Bilateral	8 (12.1)	16 (43.2)		6 (10.0)	9 (20.9)	
Pneumonia	19 (28.8)	27 (73.0)	<0.0001^*^	24 (40.0)	39 (90.7)	<0.0001^*^
Intubation	19 (28.8)	28 (75.7)	<0.0001^*^	14 (23.3)	21 (48.8)	0.013^*^
Tracheostomy	5 (7.6)	23 (62.2)	<0.0001^*^	2 (3.3)	16 (37.2)	<0.0001^*^
MMSE	25.0 [16.0–29.0]	9.0 [0.0–22.0]	<0.0001^*^	18.5 [12.0–24.5]	10.0 [0.0–19.0]	0.001^*^
BBS	28.5 [5.0–50.0]	2.0 [0.0–12.0]	<0.0001^*^	5.0 [3.0–44.5]	1.0 [0.0–6.5]	<0.0001^*^
MBSImP-oral	8.0 [5.0–12.0]	16.0 [13.0–20.0]	<0.0001^*^	10.0 [7.0–12.5]	15.0 [11.0–20.0]	<0.0001^*^
MBSImP-pharyngeal	8.0 [5.0–11.0]	13.0 [11.0–15.0]	<0.0001^*^	7.0 [5.0–10.0]	12.0 [9.0–16.0]	<0.0001^*^
PAS	8.0 [6.0–8.0]	8.0 [8.0–8.0]	<0.0001^*^	8.0 [6.0–8.0]	8.0 [8.0–8.0]	<0.0001^*^
MASA	156.0 [139.0–174.0]	94.0 [75.0–114.0]	<0.0001^*^	153.5 [140.5–166.5]	98.0 [75.0–119.5]	<0.0001^*^
EAT-10	38.0 [20.0–40.0]	40.0 [40.0–40.0]	<0.0001^*^	38.0 [25.5–40.0]	40.0 [40.0–40.0]	<0.0001^*^
FOIS	1 [1–2]	1 [1–1]	0.001^*^	1 [1–1]	1 [1–1]	0.002^*^
FAC	2 [0–3]	0 [0–0]	<0.0001^*^	0 [0–3]	0 [0–0]	0.022^*^
MBI	50.0 [20.0–78.0]	2.0 [0.0–39.0]	<0.0001^*^	27.0 [9.0–72.5]	5.0 [0.0–18.0]	<0.0001^*^
mRS (≥3)	64 (97.0)	37 (100.0)	0.285	58 (96.7)	41 (95.3)	0.733

*Values are given as number (%), means ± SD, or median [interquartile range]. Chi-squared test, Student t-test, or Mann–Whitney test was performed to compare between groups, and*

* *p <0.05 is used for statistical significance*.

*BMI, body mass index; NIHSS, National Institutes of Health Stroke Scale; MMSE, Mini-Mental State Examination; BBS, Berg Balance Scale; MBSImP, Modified Barium Swallow Impairment Profile; PAS, Penetration-Aspiration Scale; MASA, Mann Assessment of Swallowing Ability; EAT-10, Eating Assessment Tool; FOIS, Functional Oral Intake Scale; FAC, Functional Ambulatory Category; MBI, Modified Barthel Index; mRS, modified Rankin Scale*.

### Analysis of Single-Nucleotide Polymorphisms and Statistical Association With Nil per Mouth Status

Association of NPM status and genetic polymorphisms with analysis from three gene models (additive, dominant, and recessive) showed that the *rs4532* polymorphism showed a trend to increase the risk of NPM in the elderly group, both in the additive model (OR, 2.94; 95% CI, 1.17–7.37) and in the dominant model (OR, 2.93; 95% CI, 1.04–8.23) (Table 2), but with *p*-values not reaching levels of significance after multiple testing correction (*p* < 0.05/8 = 0.006) (42). In the young group, such potential association was not present (Table 2).

**Table 2:** Analysis of genetic polymorphisms and statistical association with dysphagia stratified by age.

	**HWE *p*-value**	**MAF**	**Major/minor allele**	**Age** **<** **65 (*****n*** **=** **103)**			**Age** **≥** **65 (*****n*** **=** **103)**		
				**Additive**	**Dominant**	**Recessive**	**Additive**	**Dominant**	**Recessive**
				**OR (95% CI)**	**OR (95% CI)**	**OR (95% CI)**	**OR (95% CI)**	**OR (95% CI)**	**OR (95% CI)**
*rs4532*	0.363	0.11	T/C	0.65 (0.25–1.67)	0.67 (0.25–1.81)	N/A	2.94 (1.17–7.37)^*^	2.93 (1.04–8.23)^†^	N/A
*rs165599*	0.178	0.44	A/G	0.76 (0.42–1.38)	0.96 (0.39–2.31)	0.41 (0.13–1.35)	1.31 (0.71–2.38)	1.35 (0.56–3.23)	1.49 (0.51–4.33)
*rs429358*	0.499	0.46	T/C	1.67 (0.64–4.34)	1.67 (0.64–4.34)	N/A	0.62 (0.27–1.46)	0.53 (0.21–1.37)	1.41 (0.09–23.10)
*rs4251961*	0.404	0.08	T/C	1.47 (0.56–3.86)	1.75 (0.61–5.00)	N/A	1.19 (0.41–3.50)	1.00 (0.29–3.38)	N/A
*rs1800497*	0.405	0.39	G/A	1.18 (0.64–2.17)	1.35 (0.56–3.28)	1.08 (0.36–3.27)	1.06 (0.59–1.90)	1.21 (0.54–2.69)	0.86 (0.26–2.82)
*rs4680*	0.889	0.25	G/A	0.94 (0.51–1.75)	0.78 (0.34–1.80)	1.48 (0.37–5.89)	1.22 (0.61–2.42)	1.23 (0.56–2.69)	1.42 (0.19–10.46)
*rs6265*	0.030	0.46	C/T	1.14 (0.66–1.98)	1.69 (0.66–4.32)	0.86 (0.34–2.16)	1.10 (0.66–1.83)	1.16 (0.51–2.62)	1.13 (0.46–2.81)
*rs6280*	0.059	0.29	T/C	1.16 (0.59–2.30)	1.67 (0.74–3.77)	N/A	1.21 (0.63–2.34)	1.03 (0.47–2.27)	2.97 (0.52–17.04)
*rs7412*	0.374	0.06	C/T	0.48 (0.09–2.45)	0.48 (0.09–2.45)	N/A	2.38 (0.78–7.29)	2.38 (0.78–7.29)	N/A

*N/A, Unable to assess due to small numbers of patients in recessive group*.

*HWE, Hardy–Weinberg equilibrium (p-value; control); MAF, minor allele frequency*.

*M, Major allele; m, Minor allele*.

Additive; MM (Reference) vs. Mm + 2*mm (assumption: dose-response, MM < Mm < mm).

*Dominant; MM (Reference) vs. Mm + mm*.

*Recessive; MM + Mm (Reference) vs. mm*.

* *p = 0.022,*

† *p = 0.041*.

### Swallowing Outcome Measures According to *rs4532* Phenotype

**Figure 1:**
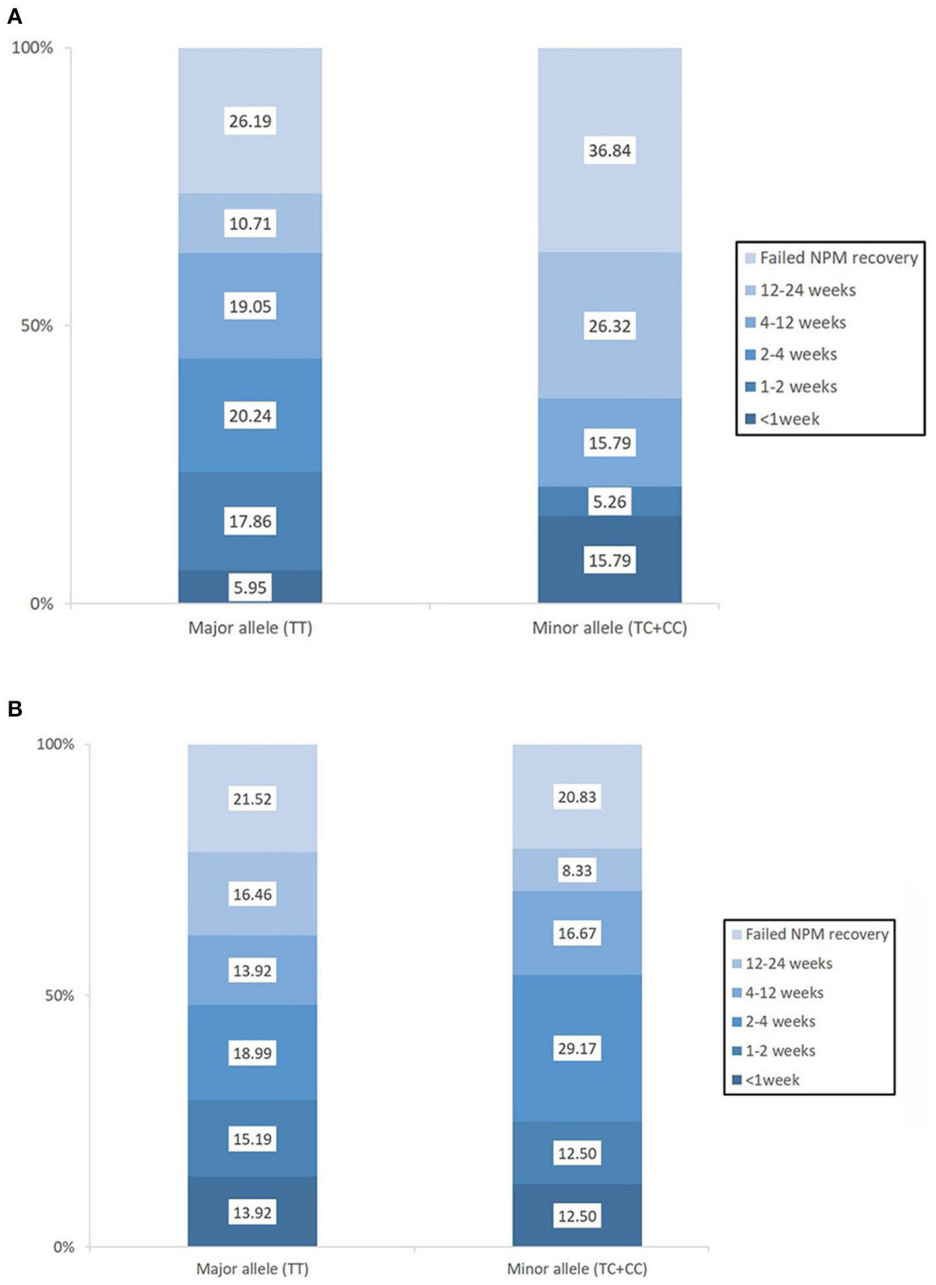
The proportion of patients who recovered from nil per mouth status within the first 3 months post-stroke according to the presence of the *rs4532* major and minor allele in those aged ≥65 **(A)** and in those aged <65 **(B)**.

**Figure 2:**
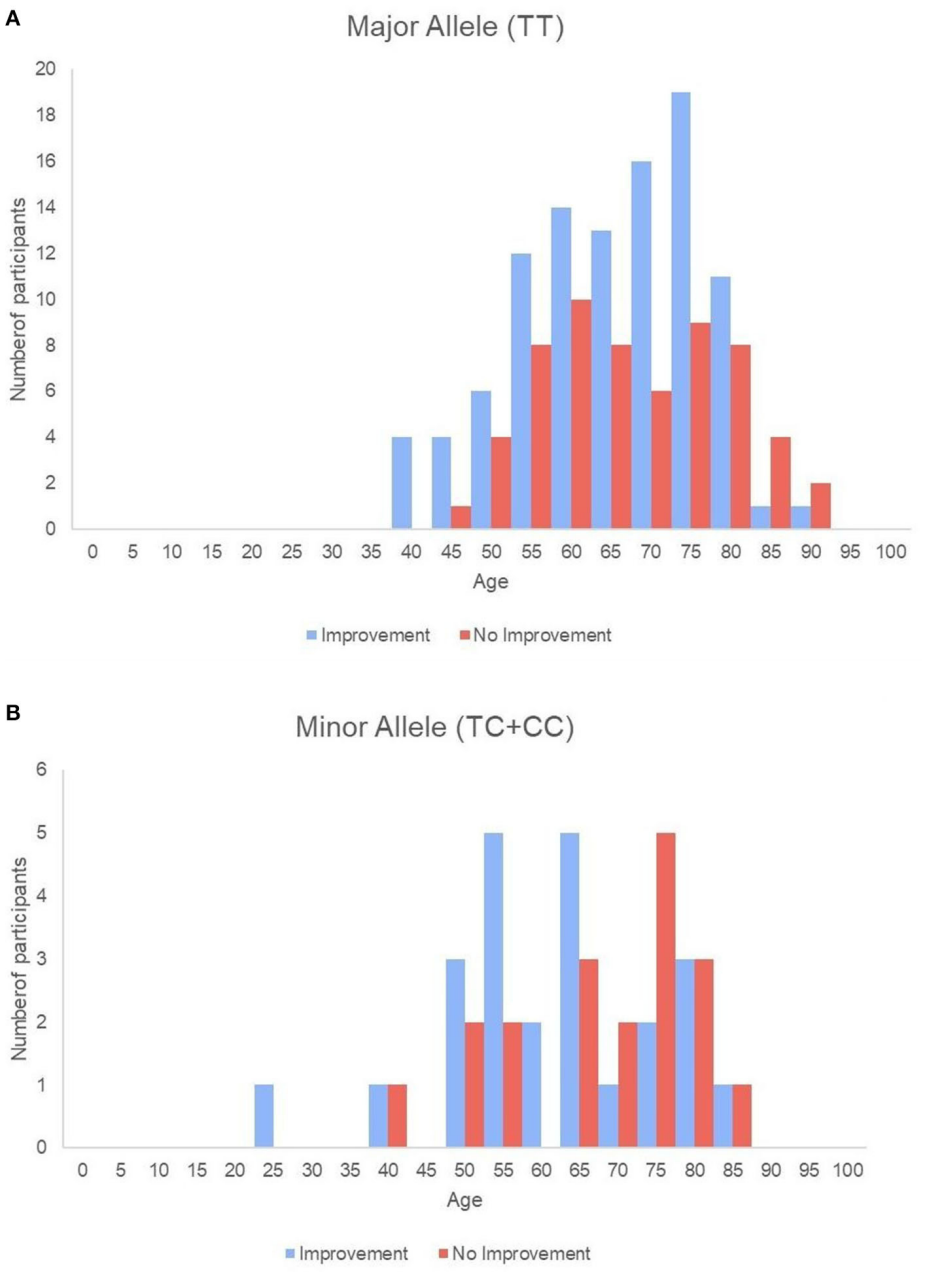
Histogram of all single subjects with or without improvement sorted by age in the major allele **(A)** and the minor allele **(B)** groups.

Baseline swallowing and functional parameters were reanalyzed according to *rs4532* phenotype within each age group. The proportion of NPM status by *rs4532* major (TT) and minor (CT+CC) allele in each age groups are presented in Figure 1. In the elderly group, those with the major allele showed significantly a higher proportion of those who recovered from NPM by 12 weeks post-stroke than those with the minor allele (63.1 vs. 36.8%, *p* = 0.036). However, in the young group, such outcome differences were not observed between the major and minor allele groups (62.0 vs. 70.84%, *p* = 0.856). When all subjects were plotted by age in a continuous scale (Figure 2), the distribution of those with or without improvement from NPM was similar in the major allele group. In the minor allele group, however, majority of the subjects with poor recovery were distributed in the elderly.

Further detailed analysis of baseline parameters showed no major differences in stroke severity or swallowing performance between those with the major or minor allele of *rs4532* within each age group, though significantly more of the elderly with the major allele were tracheostomy-free (Supplementary File 2).

**Figure 3:**
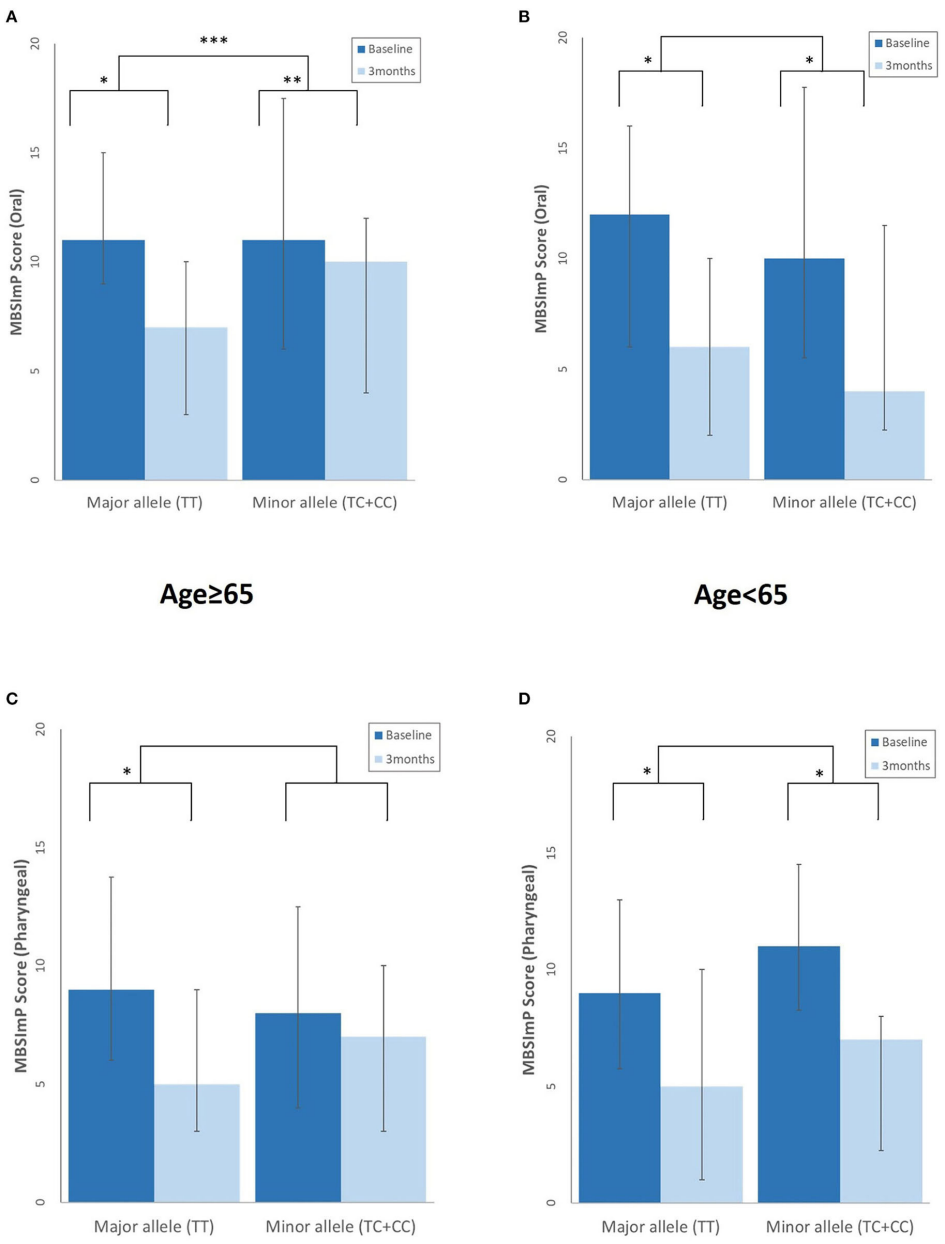
Differences in the degree of swallowing improvement at 3 months post-stroke between those with the *rs4532* major and minor allele in the modified Barium Swallow Impairment Profile (MBSImP) oral **(A,B)**, and pharyngeal component **(C,D)** in the aged ≥65 and <65 groups, with lower scores indicating better function. *p <0.001, within-group differences at baseline and at 3 months, Wilcoxon signed rank test. **p < 0.01, within-group differences at baseline and at 3 months, Wilcoxon signed rank test. ***p < 0.05, between-group differences, Mann–Whitney *U*-test.

At 3 months, the elderly with the major *rs4532* allele showed a greater degree of swallowing improvement of the oral stages as assessed by the MBSImP than those with the minor alleles. Although such similar trends were also observed in the pharyngeal component with the major allele group showing significant improvement at 3 months, it did not reach statistical significance when compared with the minor allele groups (Figure 3). In the young-age group, such intergroup differences in the degree of improvement were not observed, with both the major and minor allele groups showing a similar degree of improvement at 3 months.

### Univariable and Multivariable Analysis of Predictors of Dysphagia

A final multivariable analysis model that included age and gender as fixed variables were calculated in both age groups. Because the multiple inheritance models (additive, dominant, and recessive) showed an increased risk of association between the *rs4532* polymorphism and poor outcome with the persistence of NPM status in the elderly group, this variable was also included in the final multivariable model of the elderly.

A final model that included the presence of the *rs4532* polymorphism (OR, 10.00; 95% CI, 1.87–53.47) together with the presence of aspiration pneumonia (OR, 40.95; 95% CI, 7.98–210.29) within the first 3 months revealed high accuracy levels, with an area under receiver operating characteristic curve (AUROC) of 0.86 (95% CI, 0.79–0.93). The effect of *rs4532* was still present in the logistic regression model even for the elderly group even with the adjustment of stroke severity as assessed by the NIHSS, stroke type (i.e., hemorrhage), and low truncal control (i.e., BBS), with AUROC of 0.82 (95% CI, 0.67–0.92), 0.72 (95% CI, 0.54–0.90), and 0.71 (95% CI, 0.54–0.89), respectively (Table 3).

**Table 3:** Multivariable analysis in the age ≥65 group, with fixed variables of *rs4532* polymorphism, age, and sex.

**Variables**	**Model 1**	**Model 2**	**Model 3**	**Model 4**	**Model 5**	**Model 6**	**Model 7**
*rs4532*	10.00 (1.87–53.47)	3.17 (1.21–8.32)	2.05 (0.77–5.49)	2.54 (0.86–747)	2.18 (1.11–7.12)	3.81 (1.35–10.74)	2.77 (0.95–8.04)
Age	1.12 (1.01–1.23)	1.08 (0.99–1.17)	1.13 (1.03–1.24)	1.08 (0.99–1.17)	1.07 (0.99–1.15)	1.06 (0.98–1.14)	1.05 (0.92–1.14)
Sex	1.07 (0.35–3.25)	1.60 (0.63–4.10)	1.47 (0.52–4.14)	1.29 (0.51–3.22)	1.44 (0.57–3.61)	1.75 (0.68–4.52)	1.41 (0.57–3.50)
Pneumonia	40.95 (7.98–210.29)						
NIHSS (>14)		2.92 (1.23–6.93)					
Tracheostomy			25.2 (4.68–135.5)				
Intubation				3.71 (1.48–9.29)			
Hemorrhage					1.26 (0.48–3.25)		
BBS <21						3.40 (1.21–9.58)	
MMSE <18							1.92 (0.82–4.52)
AUROC	0.86 (0.79–0.93)	0.82 (0.67–0.92)	0.81 (0.68–0.97)	0.72 (0.62–0.82)	0.72 (0.54–0.90)	0.71 (0.54–0.89)	0.67 (0.49–0.86)

*Values are given as odds ratio (95% confidence interval)*.

*NIHSS, National Institutes of Health Stroke Scale; MMSE, Mini-Mental State Examination; BBS, Berg Balance Scale; AUROC, area under receiver operating characteristic curve*.

In contrast, in the young-age group, a final model that included variables of a prior history of intubation within the first 2 weeks (OR, 10.72; 95% CI, 3.73–30.79), along with increased age and gender, predicted poor recovery with the highest AUROC of 0.81 (95% CI, 0.73–0.90). Though tracheostomy insertion and bilateral brain lesions with episodes of aspiration pneumonia or high NIHSS scores (>14) were shown to be still valid risk factors in the multivariable analysis, they revealed only modest levels of accuracy (Table 4).

**Table 4:** Multivariable analysis in the age <65 group, with fixed variables of age and sex.

**Variables**	**Model 1**	**Model 2**	**Model 3**	**Model 4**	**Model 5**	**Model 6**	**Model 7**	**Model 8**
Age	1.11 (1.03–1.20)	1.04 (0.97–1.11)	1.07 (0.99–1.16)	1.05 (0.99–1.12)	1.07 (1.00–1.14)	1.07 (1.00–1.14)	1.07 (1.00–1.14)	1.02 (0.96–1.09)
Sex	0.54 (0.20–1.44)	0.40 (0.15–1.05)	1.25 (0.38–4.12)	0.74 (0.28–1.94)	0.59 (0.24–1.47)	0.46 (0.18–.1.15)	0.52 (0.21–1.29)	0.61 (0.24–1.52)
Intubation	10.72 (3.73–30.79)							
Pneumonia		6.80 (2.65–17.42)						
Tracheostomy			22.94 (6.54–80.47)					
BBS (<21)				6.42 (2.44–16.92)				
Hemorrhage					2.62 (1.02–6.71)			
Bilateral						6.52 (2.29–18.61)		
NIHSS (>14)							3.53 (1.45–8.57)	
MMSE (<18)								3.80 (1.53–9.47)
AUROC	0.81 (0.73–0.90)	0.77 (0.68–0.87)	0.76 (0.57–0.94)	0.76 (0.66–0.85)	0.74 (0.57–0.90)	0.74 (0.64–0.85)	0.73 (0.63–0.83)	0.71 (0.61–0.82)

*Values are given as odds ratio (95% confidence interval)*.

*BBS, Berg Balance Scale; NIHSS, National Institutes of Health Stroke Scale; MMSE, Mini-Mental State Examination; AUROC, area under receiver operating characteristic curve*.

## Discussion

According to our study, elderly patients were more susceptible to the effects of the SNPs and among the various SNPs, variation of *rs4532* related to dopamine was shown to be potentially associated to less favorable outcomes in post-stroke swallowing recovery. Also, the elderly with the minor allele showed poorer improvement at follow-up in the swallowing parameters. In consideration of other clinical variables from the multivariable regression model, those with the minor allele of DRD1 (*rs4532*), with a previous episode of aspiration pneumonia showed the greatest ORs in poor post-stroke swallowing outcome in the elderly age group. By contrast, clinical variables that included prior intubation showed the greatest ORs for poor outcome in the young age group and gene effects were not shown to adversely influence recovery. The results of our study suggest that the effects of gene may amplify in those with elderly stroke patients and lead to adverse outcomes in swallowing with persistence of NPM at 3 months post onset.

Among the many factors that influence stroke outcome, age is a crucial prognostic factor. In the Copenhagen Stroke Study, an increase in age resulted in a decrease in the BI score gains (43). In addition, in the Framingham Study, disability was more frequently noted when the age of stroke onset was higher (44). Likewise, the elderly group showed poor recovery in MBI and FAC scores at 3 months. In a similar manner, dysphagia outcomes may also be influenced by age. A recent study showed that individuals over 65 years old had an increased risk of poor swallowing outcomes (22). With aging, the protective reflex may be impaired, which functions as a preventive mechanism against aspiration in healthy populations (45). Moreover, presbyphagia may manifest as decreased tongue movement, bolus propulsion, and poor upper esophageal sphincter opening (46).

There have been a number of studies conducted on age-dependent effects on gene. ApoE ε2 was reported as a risk factor of cerebral infarction in Japanese populations, but the association between ApoE ε2 and stroke occurrence was only prominent in individuals aged 70 years old and older (18). Similarly, ApoE ε2 carrier status was associated with hemorrhagic stroke in individuals aged 60 years old and older in a Bangladeshi study (47). One study focusing on the COMT (*rs4680*) gene's association with cognitive performance in non-demented adults revealed that the gene's effect on visuospatial ability was seen only in middle-aged (35–45 years) individuals (19). Another study assessed the association of the BDNF (*rs6265*) gene on stroke rehabilitation outcomes, revealing significant differences in functional parameters such as MBI and mRS scores between individuals under and over the age of 55 years (15). Furthermore, genetic variability in the old may affect cognitive performance more strongly than in the young (14). Consistent with these past studies, our results indicate that age-dependent effects may potentially influence post-stroke swallowing recovery with possible association with the dopamine-related gene (*rs4532*).

Dopamine is one of the neurotransmitters closely related to swallowing (48). In a previous study, dopamine agonist (apomorphine) improved swallowing in the early stages of Parkinson's disease (49). Furthermore, levodopa, which is the precursor to dopamine, norepinephrine, and epinephrine, may sometimes be effective in dysphagia in Parkinson's disease (50, 51). COMT and dopamine receptor genes (DRD1, DRD2, and DRD3) are involved in dopamine neurotransmission. Of these, the Met allele of COMT, not dopamine receptor genes, is known to be significantly associated with motor recovery in stroke patients (25).

The DRD1 gene encodes the D1 dopamine receptor subtype, and *rs4532* is an SNP of DRD1. The DRD1 C allele is reported to be associated with increased dopamine transmission in the brain (52). Moreover, those with homozygous DRD1 C alleles are known to have better D1 receptor efficiency than those with the T allele (53). Given the minor C allele's role in increased D1 receptor efficiency and therefore increased dopamine transmission, those with the minor C allele may be expected to show better outcomes. However, in this study, the minor C allele of *rs4532* was associated with an increased risk of poor outcome in the older group. Even after adjustment of other confounding factors, the presence of the minor *rs4532* allele showed a 10-fold higher risk of developing dysphagia in the elderly group. Of interest was the fact that such association was not present with other SNPs related to DRD2 or DRD3 or those related to COMT.

Previous studies (14, 54) have hypothesized that the efficacy of dopamine signaling on functional outcomes may follow an inverted U-shape. In other words, functional outcomes may be greatest at optimum dopamine signaling efficacy, and if the efficacy is increased or decreased compared with the optimum level functional outcomes may instead decline. In the young, the efficacy of dopamine signaling is near its optimal level and may adapt to damaged dopamine pathways efficiently, incurring little differences in functional outcomes between major and minor allele groups. On the other hand, in elderly individuals, even minor damage to the dopamine pathways may influence the efficacy of dopamine. In addition, excessive dopamine release during the acute stages of stroke has been proven to be more toxic, leading to worse outcomes (55) and proven more detrimental in the elderly from animal models of stroke (56). Through a similar mechanism after stroke, increased levels of dopamine may make the elderly more susceptible to the detrimental effects of excessive dopamine. Those with the DRD1 minor C allele may be at risk of too much dopamine transmission, far beyond optimal levels in the inverted U-shape curve. Therefore, in those over 65 years of age, genetic effects between the major T allele and minor C allele may have magnified the differences in swallowing, resulting in different NPM states at 12 weeks, as seen in our study.

Unlike their counterparts, individuals under 65 years old seemed not to be affected by the presence of the *rs4532* polymorphism. Instead, clinical parameters such as BMI, NIHSS, intubation history, and bilateral stroke lesions, seemed to significantly increase the risk of NPM. These findings are consistent with those of previous studies, whereby old age, male sex, bilateral stroke involvement, initial NIHSS score, dysarthria, and intubation history are already known to lead to poor outcomes in post-stroke dysphagia (41, 57, 58). In the final multivariable analysis, the presence of intubation seemed to predict NPM status with higher accuracy than other variables including the NIHSS. Intubation is more often performed in the most severely neurologically debilitated, explaining the high predictability in NPM outcomes. However, iatrogenic effects resulting in long-lasting swallowing dysfunction have been suggested after intubation and also after tracheostomy insertion, even in those without neurological deficits. Therefore, the detrimental mechanisms of intubation *per se* in light of swallowing outcomes need to be further assessed in future studies (59).

There are certain limitations to this study. First, this was a secondary analysis from a prospective study, and although the results showed possible associations, these did not reach the desired statistical significance after multiple testing. Nevertheless, the elderly with *rs4532* polymorphism showed poorer recovery in the follow-up parameters with possible effect in the multivariable logistic regression model even after adjustment of other covariates. Second, despite the positive trend, the sample size of our study may not have been large enough to meet the stringent *p*-value criterion, though for the primary objective of the trial, power analysis was based on a prognosis model (20). Our sample size is similar to previous studies on SNP polymorphism and stroke outcome [Cramer et al. (12) *n* = 255, Kim et al. (25) *n* = 74] and is one of the few prospective SNP studies with the largest sample of stroke subjects with full follow-up of data on the instrumental swallowing tests. Due to the potential impact of this SNP on post-stroke dysphagia outcome in the elderly, our results, though exploratory in nature, deserve attention and need to be replicated by larger scale prospective studies. Third, due to the secondary analysis design of the study, we stratified the group into two age groups based on definitions made from previous literature (21, 22). However, to fully elucidate the influence of age and genetic polymorphisms, future prospective studies using a non-linear continuous analysis of age are warranted to further investigate the age-related outcomes. Fourth, NPM at 3 months post-stroke onset was selected as the primary outcome of this study. Using NPM as endpoint may identify only the most serious cases and could lead to bias. However, NPM after stroke is closely associated with respiratory infection after stroke, which increases mortality (60), and reflects accurately the level of swallowing disability. In fact, duration of impaired oral intake is known as the decisive parameter of swallowing recovery as suggested by guidelines (61). Therefore, we deemed NPM at 3 months as a valid primary outcome parameter. Another point to address was the relationship between the dopamine and function. Though previous studies (14, 54) have suggested an inverted U-shape in dopamine signaling efficacy, these are yet speculative in nature. Though theoretically sound, the role of dopamine and its relationship with the inverted U-shape response warrants future studies. More studies on dopamine signaling efficacy are needed to further support our results. Finally, while BDNF was associated with improved swallowing outcome in stroke patients in a previous study, DRD1 was shown to be involved with poor outcome in this study, raising the question which SNP is the critical biomarker. In reality, no single SNP would be able to explain for differences in motor recovery across different individuals. As proposed in past studies (62), different genes may exert different influences. Also, a close interaction between BDNF and genes related to dopamine neurotransmission have been suggested by past studies through COMT polymorphisms and affect plasticity process (63, 64). Future studies on SNPs and post-stroke swallowing recovery focusing on the BDNF and DRD1 interactions may be needed.

In line with previous studies that the same gene polymorphisms may exert different influences in stroke recovery in the elderly (54, 65), our study suggests that the presence of the DRD1 minor C allele may potentially play a role in increasing the risk of the poor swallowing outcome at 12 weeks post-onset in those over the age of 65. To the best of our knowledge, this is one of the first studies to suggest that with increased age, effects of genetic variations related to dopamine may become prominent (14) with age after stroke and that the elderly may be more vulnerable to the effects of SNPs and show less favorable outcomes in swallowing recovery.

## Conclusions

The presence of the DRD1 polymorphism may play a role in poor swallowing recovery after stroke. Such SNP variations may potentially serve to be as useful biomarkers in predicting dysphagia recovery in elderly stroke patients. The role of SNP in the elderly stroke subjects is a topic that needs to be rigorously addressed in future studies.

## Data Availability Statement

The datasets presented in this article are not readily available due to confidentiality agreements, and supporting data can only be made available to bona fide researchers subject to a non-disclosure agreement. Details of the data and how to request access should be directed to the corresponding author.

## Ethics Statement

The studies involving human participants were reviewed and approved by institutional review board of Catholic Medical Center. The patients/participants provided their written informed consent to participate in this study.

## Author Contributions

G-YP and SI were responsible for conceptualization, methodology, and study administration. SI performed data curation and analysis. H-YP and SI wrote the original draft, which was revised and edited by HO, T-WK, and YK. All authors contributed to the article and approved the submitted version.

## Conflict of Interest

The authors declare that the research was conducted in the absence of any commercial or financial relationships that could be construed as a potential conflict of interest.
