# Supplementary material for: Potential Prognostic Impact of Dopamine Receptor D1 (rs4532) Polymorphism in Post-stroke Outcome in the Elderly
Source: Front Neurol. 2021 Jun 30;12:675060. doi: 10.3389/fneur.2021.675060 (PMC8277925; doi:10.3389/fneur.2021.675060)
Supplement: Supplementary file 1 [file Data_Sheet_1.docx]

**Supplementary Materials**

Supplementary file 1. Baseline clinical demographics of patients categorized by age

Supplementary file 2. Baseline swallowing parameters according to *rs4532* genotypes of the patients categorized by age groups

Supplementary file 1. Baseline clinical demographics of patients categorized by age

|  | Age < 65 years | Age ≥ 65 years | P-value |
| --- | --- | --- | --- |
|  | (n = 103) | (n = 103) |  |
| Sex (male) | 69 (67.0) | 67 (65.1) | 0.769 |
| BMI | 22.76 ± 2.94 | 22.09 ± 3.55 | 0.141 |
| NIHSS | 13.3 ± 7.4 | 12.9 ± 7.2 | 0.761 |
| Stroke type |  |  | < .001^*^ |
| Infarction | 45 (43.7) | 74 (71.8) |  |
| Hemorrhage | 54 (52.4) | 27 (26.2) |  |
| Both | 4 (3.9) | 2 (1.9) |  |
| Lesion location |  |  | 0.442 |
| Supratentorial | 73 (70.9) | 76 (73.8) |  |
| Infratentorial | 24 (23.3) | 25 (24.8) |  |
| Multiple | 6 (5.8) | 2 (1.9) |  |
| Lesion side |  |  | 0.248 |
| Right | 33 (32.0) | 40 (38.8) |  |
| Left | 46 (44.7) | 48 (46.6) |  |
| Bilateral | 24 (23.3) | 15 (14.6) |  |
| Diabetes | 37 (35.9) | 48 (46.6) | 0.120 |
| Hypertension | 69 (67.0) | 80 (77.7) | 0.087 |
| Alcohol | 37 (35.9) | 31 (30.1) | 0.374 |
| Smoking | 32 (31.1) | 30 (29.1) | 0.761 |
| Pneumonia | 46 (44.7) | 63 (61.2) | 0.018^*^ |
| Intubation | 47 (45.6) | 35 (34.0) | 0.117 |
| Tracheostomy | 28 (27.2) | 18 (17.5) | 0.132 |
| MMSE | 22 [8-27] | 17 [7-24] | 0.012^*^ |
| BBS | 19 [2-47] | 4 [0-30] | 0.018^*^ |
| NPM at ≥12 weeks | 37 (35.9) | 43 (41.0) | 0.391 |
| MBSImP© - oral | 11 [6-14] | 12 [9-16] | 0.105 |
| MBSImP©- pharyngeal | 10 [6-13] | 9 [6-13] | 0.769 |
| PAS | 8 [7-8] | 8 [8-8] | 0.092 |
| MASA | 139 [104-169] | 139 [102-155] | 0.585 |
| EAT-10 | 40 [28-40] | 40 [32-40] | 0.423 |
| FOIS | 1 [1-1] | 1 [1-1] | 0.426 |
| FAC | 0 [0-3] | 0 [0-2] | 0.037 |
| MBI | 39 [1-64] | 15 [3-56] | 0.203 |
| mRS (≥3) | 101 (98.1) | 99 (96.1) | 0.683 |

Values are given as number (%), means ± SD, or median [interquartile range].

BMI: body mass index; NIHSS: National Institutes of Health Stroke Scale; MMSE: Mini-Mental State Examination; BBS: Berg Balance Scale; NPM: nil per mouth; MBSImP^©^: Modified Barium Swallow Impairment Profile; PAS: Penetration-Aspiration Scale; MASA: Mann Assessment of Swallowing Ability; EAT-10: Eating Assessment Tool; FOIS: Functional Oral Intake Scale; FAC: Functional Ambulatory Category; MBI: Modified Barthel Index; mRS: modified Rankin Scale.

^*^ P-value < 0.05 are used for statistical significance.

Supplementary file 2. Baseline swallowing parameters according to *rs4532* genotypes of the patients categorized by age groups

|  | Age < 65 (n = 103) | | |  | Age ≥ 65 (n = 103) | | |  |
| --- | --- | --- | --- | --- | --- | --- | --- | --- |
|  | TT  (N = 79) | CT  (N = 23) | CC  (N = 1) | p-value | TT  (N = 84) | CT  (N = 16) | CC  (N = 3) | p-value |
| Age | 54.7 ± 6.7 | 54.0 ± 7.5 | 25.0 | 0.059 | 73.2 ± 5.6 | 74.8 ± 5.2 | 72.0 ± 7.0 | 0.589 |
| NIHSS | 13.7 ± 7.1 | 11.6 ± 8.3 | 18.0 | 0.389 | 12.8 ± 7.4 | 12.8 ± 6.5 | 17.7 ±3.5 | 0.472 |
| Stroke type |  |  |  | 0.532 |  |  |  | 0.482 |
| Infarction | 35 (44.3) | 9 (39.1) | 1 (100) |  | 63 (75.0) | 9 (56.2) | 2 (66.7) |  |
| Hemorrhage | 42 (53.2) | 12 (52.2) | 0 (0.0) |  | 19 (22.6) | 7 (43.8) | 1 (33.3) |  |
| Both | 2 (2.5) | 2 (8.7) | 0 (0.0) |  | 2 (2.4) | 0 (0.0) | 0 (0.0) |  |
| Aspiration pneumonia | 34 (43.0) | 12 (52.2) | 0 (0.0) | 0.492 | 53 (63.1) | 8 (50.0) | 2 (66.7) | 0.604 |
| Intubation | 37 (46.8) | 9 (39.1) | 1 (100) | 0.443 | 27 (32.1) | 6 (37.5) | 2 (66.7) | 0.440 |
| Tracheostomy | 23 (29.1) | 5 (21.7) | 0 (0.0) | 0.648 | 11 (13.1)^*^ | 6 (37.5)^*^ | 1 (33.3) | 0.048^*^ |
| MBSImP^©^- oral | 12.0  [6.0-16.0] | 10.0  [6.0-15.5] | 17.0  [17.0-17.0] | 0.380 | 11.0  [9.0-15.0] | 10.0  [6.0-13.5] | 18.0  [15.0-22.0] | 0.168 |
| MBSImP^©^-pharyngeal | 9.0  [6.0-13.0] | 11.0  [8.5-14.0] | 9.0  [9.0-9.0] | 0.219 | 9.0  [6.0-13.5] | 8.0  [4.5-11.5] | 8.0  [4.0-12.0] | 0.504 |
| PAS | 8.0  [7.0-8.0] | 8.0  [8.0-8.0] | 8.0  [8.0-8.0] | 0.084 | 8.0  [8.0-8.0] | 8.0  [5.0-8.0] | 8.0  [8.0-8.0] | 0.05 |
| EAT-10 | 40.0  [24.5-40.0] | 40.0  [39.0-40.0] | 40.0  [40.0-40.0] | 0.282 | 40.0  [33.0-40.0] | 40.0  [22.0-40.0] | 40.0  [36.0-40.0] | 0.886 |
| FOIS | 1.0  [1.0-1.0] | 1.0  [1.0-1.0] | 1.0  [1.0-1.0] | 0.900 | 1.0  [1.0-1.0] | 1.0  [1.0-2.0] | 1.0  [1.0-1.0] | 0.297 |
| BBS | 15.0  [0.0-47.0] | 23.0  [3.0-42.5] | 19.0  [19.0-19.0] | 0.984 | 4.0  [0.0-28.0] | 16.0  [1.5-47.5] | 0.0  [0.0-1.5] | 0.149 |

Values are given as means ± SD, number (%), or median [interquartile range].

NIHSS: National Institutes of Health Stroke Scale; MBSImP^©^: Modified Barium Swallow Impairment Profile; PAS: Penetration-Aspiration Scale; EAT-10: Eating Assessment Tool; FOIS: Functional Oral Intake Scale.

Kruskal Wallis was done for numerical values and chi-square test was done for categorical values. ^*^ P-value < 0.05 are used for statistical significance.
